# Photoinactivation of Multidrug-Resistant *mcr-1*-Positive *E. coli* Using PCPDTBT Conjugated Polymer Nanoparticles under White Light

**DOI:** 10.1021/acsabm.4c01049

**Published:** 2024-10-18

**Authors:** Cynthia S. A. Caires, Thalita H. N. Lima, Rafael C. Nascimento, Leandro O. Araujo, Laís F. Aguilera, Anderson R. L. Caires, Samuel L. Oliveira

**Affiliations:** † Instituto de Física, Universidade Federal de Mato Grosso do Sul, CP 549, 79070-900 Campo Grande, MS, Brazil; ‡ Escola de Saúde, Santa Casa de Campo Grande, 79002-201 Campo Grande, MS, Brazil; § Instituto de Física de São Carlos, Universidade de São Paulo, CP 369, 13560-970 São Carlos, SP, Brazil

**Keywords:** photodynamic inactivation, mcr-1 positive Escherichia coli, antimicrobial resistance, conjugated polymer, nanoparticle, PCPDTBT

## Abstract

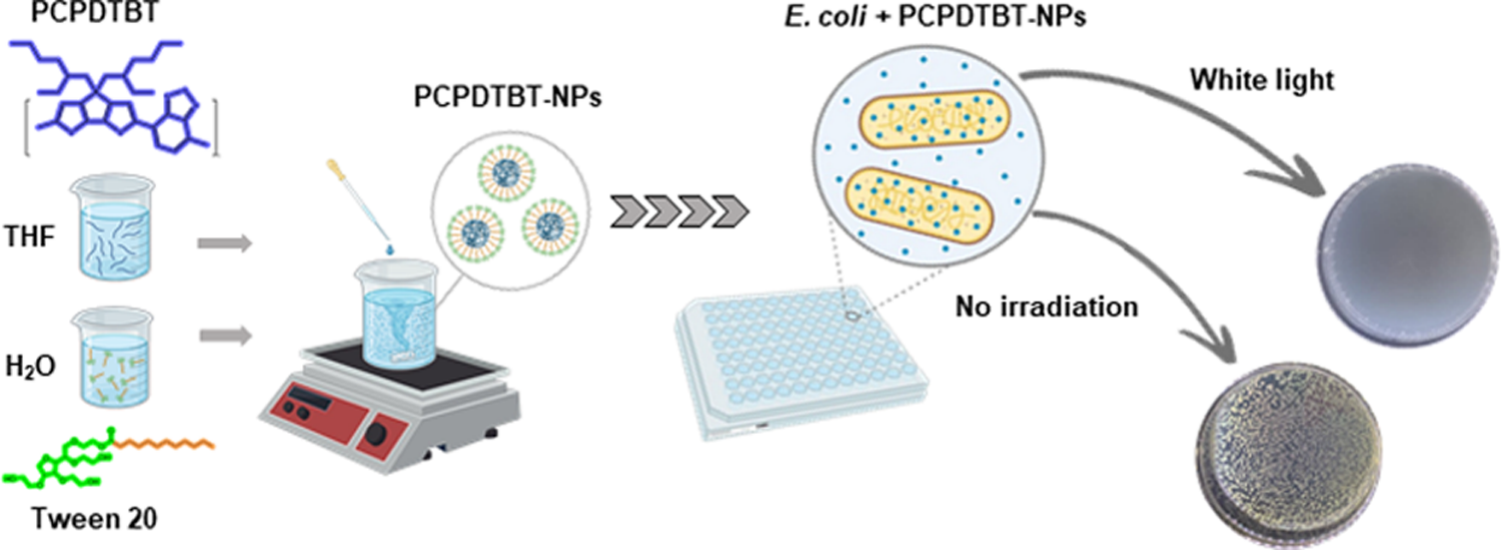

The issue of antimicrobial resistance is an escalating concern within the scope of global health. It is predicted that the existence of antibiotic-resistant bacteria might result in an estimated annual death of up to 10 million by 2050, along with possible economic losses ranging from 100 to 210 trillion. This study reports the production of poly[2,6-(4,4-bis(2-ethylhexyl)-4H-cyclopenta[2,1-b;3,4-b’]dithiophene)-*alt*-4,7(2,1,3-benzothiadiazole)] nanoparticles (PCPDTBT-NPs) by nanoprecipitation as an alternative to tackle this problem. The size, shape, and optical features of these conjugated polymer NPs were analyzed. Their efficacy as photosensitizers against nonresistant (ATCC) and multidrug-resistant *mcr*-1-positive *Escherichia coli* was assessed under white light doses of 250 and 375 J·cm^–2^. PCPDTBT-NPs inactivated both *E. coli* strains exposed to white light at an intensity of 375 J·cm^–2^, while no antimicrobial effect was observed in the group not exposed to white light. Reactive oxygen species and singlet oxygen were detected using DCFH-DA and DPBF probes, allowing the investigation of the photoinactivation pathways. This work showcases PCPDTBT-NPs as photosensitizers to eliminate multidrug-resistant bacteria through photodynamic inactivation employing visible light.

## 1 Introduction

The world is experiencing a significant rise in infection rates caused by antimicrobial resistance (AMR) and a shortage of new and effective antimicrobial medicines. This makes AMR a top-priority worldwide issue that needs to be urgently tackled.^1^ In 2019, around 1.3 million individuals globally died due to bacteria that were resistant to antibiotics.^2^

Studies indicate that deaths caused by AMR microorganisms are projected to rise, resulting in an average annual economic loss of 3 billion dollars.^3^ Nevertheless, it is imperative to recognize the global impact of the COVID-19 pandemic on the rise of antimicrobial resistance (AMR) as an outcome of the heightened demand for antibiotics required by pandemic treatments.^2^ It is critical to evaluate the impact of the extensive use of disinfectants and antimicrobial agents on the spreading of AMR during the pandemic.^4^

In 2016, Chinese scientists first found the *mcr-1* gene in samples obtained from animals, food, and even humans.^5^ After this first discovery, many other cases were recorded in various countries.^6,7^ Researchers have identified 10 variants of the *mcr* gene.^8^ Although genetic mutations of *mcr* have been documented, indicating their ongoing evolution, they have not spread globally like the original *mcr-1*(9,10) This gene enables *Escherichia coli* (*E. coli*) to develop resistance to colistin medicines mediated by plasmids that carry antibiotic resistance genes (ARGs) across bacteria, even in genetically unrelated species.^2^

Colistin, a broad-spectrum antibiotic in the polymyxin class, is employed as a final option to deal with infections caused by Gram-negative bacteria resistant to multiple medicines.^9^ It has been utilized in both human and veterinary medicine. It was primarily employed in animals to mitigate gastrointestinal diseases in pigs and poultry. Each country has its own set of restrictions related to the usage of colistin for this purpose. Finland, Iceland, and Norway do not use it, whereas Italy and Spain utilize over 20 mg per kg of animal biomass.^7^ Restrictions on the intravenous usage of colistin in humans have been implemented due to concerns regarding its nephrotoxicity and neurotoxicity.^7^ Currently, the predominant concern lies in the multidrug resistance in *E. coli*. This resistance is caused by specific strains that produce extended-spectrum β-lactamase (ESBL), which makes them immune to third and fourth-generation antibiotics such as cephalosporins and fluoroquinolones.^8^

The current situation underscores the need to enhance or create clinical methods to combat colistin-resistant bacteria. Antimicrobial photodynamic inactivation (aPDI) emerges as a valuable strategy to produce singlet oxygen and reactive oxygen species (ROS) to destroy bacteria.^11^ After photosensitizer (PS) excitation and its interaction with molecular oxygen, two possible reactions occur: charge transfer, which leads to the formation of radicals or radical ions (known as Type I reaction), and energy transfer to molecular oxygen, resulting in singlet oxygen production (known as Type II reaction). Both processes oxidize the bacteria′s cells, causing their death.^12−14^

A significant advantage of aPDI is its ability to target multiple sites, which differs from antibiotics that only act in a single site using a specific mechanism to cause bacteria death (for instance, inhibiting nucleic acid synthesis, bacterial cell wall synthesis, ribosome function, or cell membrane function).^15,16^ Consequently, it is improbable that resistance to aPDI occurs since it induces cell death by generating ROS at various sites, triggering damage to different bacterial cell structures simultaneously.^11,14,17−20^ To illustrate, recent studies have demonstrated the great potential of aPDI mechanisms to deal with *mcr-1-positive* bacteria.^21,22^

Conjugated polymer nanoparticles (CPNs) are multifunctional materials with versatility and applications across various technological domains. There is a rising focus on employing their physicochemical characteristics to develop photothermal and photodynamic strategies to overcome multidrug-resistant bacteria.^23,24^ The conjugated polymer poly[2,6-(4,4-bis(2-ethylhexyl)-4H-cyclopenta[2,1-b;3,4-b’]dithiophene)-*alt*-4,7(2,1,3-benzothiadiazole)] (PCPDTBT) has been explored as photothermal agent, alone or associated with other compounds, for a range of phototherapy applications.^25−28^ However, to our knowledge, this polymeric nanoparticle has not yet been used in aPDI as a PS, as reported here. This paper presents the synthesis, characterization, and evaluation of PCPDTBT nanoparticles as photosensitizers for aPDI, detailing their potential efficacy and mechanism of action against multidrug-resistant *E. coli*.

## 2 Materials and Methods

### 2.1 Photosensitizer

**Figure 1:**
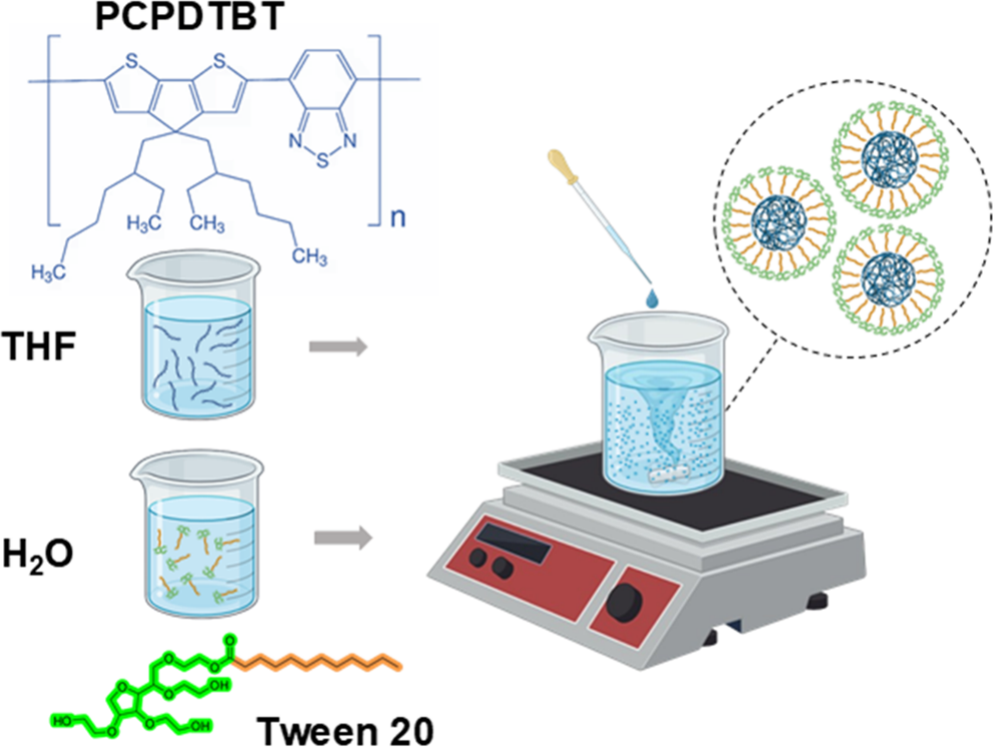
Schematic diagram of the PCPDTBT nanoparticles production by nanoprecipitation method.

Nanoparticles of PCPDTBT (Sigma-Aldrich) nanoparticles were produced by nanoprecipitation, as outlined in Figure 1.^29^ PCPDTBT was dissolved in tetrahydrofuran (THF) at a concentration of 0.23 mg·mL^–1^. The resultant solution was added dropwise to a 10 mL aqueous solution of polysorbate 20 (Tween 20, Quimesp Química Ltd., Brazil) at a concentration of 1.2 mg·mL^–1^ while stirring slowly. After 12 h of stirring, the water and THF evaporated and were replaced with the surfactant solution to maintain the volume of the nanosuspension at 10 mL. The concentration of PCPDTBT-NPs in the stock solution was 45.9 μg·mL ^–1^.

### 2.2 Characterization of the Photosensitizer

#### 2.2.1 Absorption and Fluorescence Measurements of PCPDTBT-NPs

UV–vis absorption spectra were acquired in the wavelength range of 250 to 900 nm using a deuterium-tungsten lamp (DH-2000, Ocean Optics), a portable spectrometer (HR4000, Ocean Optics), and optical fibers (TP 300 UV–vis, Ocean optics). Fluorescence spectra were obtained employing a 405 nm diode laser with an intensity of 74 mW·cm^–2^. Excitation delivery and fluorescence collection were performed using a y-type optical fiber (TP 300 UV–vis, Ocean optics) coupled to a portable spectrometer (HR4000, Ocean Optics) to collect the fluorescence in the 450 and 1050 nm range with the front-side configuration. The samples (20 mg·L^–1^) were measured at room temperature in a quartz cuvette (1 cm optical length).

#### 2.2.2 Scanning Electron Microscopy and Dynamic Light Scattering

The size and morphology of PCPDTBT-NPs were examined using scanning electron microscopy (SEM) with JEOL model JSM-6380LV operating at 10 kV. The PCPDTBT-NPs solution was deposited onto a 1 × 1 cm glass substrate and left to dry overnight under ambient conditions. The sample was sputter-coated with gold and mounted onto SEM holders with carbon. The SEM images were analyzed with ImageJ to determine the particle diameter distribution and morphology. The mean size of the PCPDTBT-NPs was calculated by assuming the spherical shape of 100 particles. The hydrodynamic size, polydispersity index, and ζ potential were also analyzed by dynamic light scattering (DLS) in a Zetasizer NanoZS device (Malvern Instruments Ltd.a, UK). The mean hydrodynamic diameter was obtained by measuring a solution of PCPDTBT-NPs at a concentration of 4.9 μg·mL^–1^ shortly after dilution at 25 °C. NaCl was added to the PCPDTBT-NPs samples to measure the ζ potential at a final working concentration of 9 mM.

#### 2.2.3 Photostability and Stored Stability

A nanoparticle solution at 17 μg·mL^–1^ was subjected to white light illumination from an RGB LED device to determine the photostability of PCPPDTB-NPs. The UV–vis absorption spectra in the 250 to 900 nm range were collected using a LAMBDA 265 UV/vis spectrophotometer (PerkinElmer) as a function of the time for 90 min. The nanoparticles’ photostability was tested using the maximum light dose employed in the aPDI assay (375 J·cm^–2^). The storage stability was also evaluated using two solutions of PCPPDTB-NPs at 17 μg·mL^–1^, one stored in a refrigerator at approximately 4 °C and the other at room temperature. The samples were exposed to light only during the experiments.

### 2.3 Strains

The tests used a nonmultidrug-resistant strain of *E. coli* (ATCC 25922) and a multidrug-resistant *mcr-1* positive strain (CCBH 23595) received from the Oswaldo Cruz Institute (FIOCRUZ). Both strains were preserved in Muller Hinton Broth (20% v·v^–1^ glycerol) at −70 °C. To prepare the bacterial suspension, 10 μL of each strain was added to 2 mL of Brain Heart Infusion (BHI) and incubated at 37 °C for 24 h. A turbidity standard of 0.5 McFarland was adopted to ensure the same bacterial concentration.

### 2.4 Photophysical and Photochemical Analysis

#### 2.4.1 Singlet Oxygen (^1^O_2_) Generation

The ^1^O_2_ formation was monitored using 1,3-diphenylisobenzofuran (DPBF) (Sigma-Aldrich, Brazil) as a probe.^30,31^ Measurements were performed with 2400 μL of water and 480 μL of PCPDTBT-NPs at a concentration of 45.9 μg·mL^–1^. Next, 200 μL of DPBF solution at 1 mM was introduced into the cuvette with an optical path of 10 mm, resulting in final solutions with the photosensitizer at 8.5 μg·mL^–1^ and ^1^O_2_ probe at 0.077 mM. The solutions were exposed to 625 nm irradiation with an intensity of 3.5 mW·cm^–2^ for 210 s from the RGB LED device. UV–vis absorption was used to monitor the ^1^O_2_ formation every 30 s during irradiation. The measurements were made in a LAMBDA 265 UV/vis spectrophotometer (PerkinElmer).

Red-light-induced reactive oxygen species (ROS) generated by PCPDTBT-NPs were studied using the nonfluorescent marker 2′,7′ dichlorofluorescein diacetate (DCFH-DA) (Sigma-Aldrich). This marker undergoes oxidation upon interaction with ROS and turns into a fluorescent molecule (DCF).^32^ A DCFH-DA stock solution containing 5 mM in ethanol was prepared. DCFH-DA and PCPDTBT-NPs solutions were combined, diluted with distilled water, and transferred to a quartz cuvette to obtain concentrations of 0.35 mM for DCFH-DA and 8.5 mg·L^–1^ for PCPDTBT-NPs. Exciting the samples at 470 nm and detecting emission in the 500–600 nm range made monitoring the ROS production continuously possible. The initial 10 min investigated the occurrence of ROS generation without light (chemical reaction), followed by the activation of red light to assess the ROS production under illumination (photochemical reaction).

### 2.5 Photoinactivation Assay

PCPDTBT-NPs were diluted to concentrations of 0 (negative control), 4.25, 8.5, and 17 μg·mL^–1^ in a 2 mL saline solution with *E. coli* inoculum. After adding PCPDTBT-NPs, they were agitated in a shaker (Marconi, Brazil) at 120 rpm for 60 min. The samples were divided into two groups: the nonirradiated (dark) group and the irradiated group, which was exposed to energy doses of 250 and 375 J·cm^–2^ over an irradiation period of 60 and 90 min, respectively. The energy was delivered as white light emitted by an RGB LED-based device that combined 625, 525, and 450 nm.

Both irradiated and nonirradiated groups, treated with PCPDTBT-NPs, had 200 μL of each sample concentration dispensed into a 96-well microplate. Serial dilutions were made until 1:32. The colony-forming units (CFU) were determined after 18 h of incubation at 37 °C using the spread plate method and the Plate Count Agar (PCA) as the medium. The experiments were conducted in duplicate and repeated three times to obtain CFU values for the four photosensitizer concentrations in both the irradiated and nonirradiated groups. The Origin 8.5 software was used to perform the statistical analysis. The CFU·mL^–1^ numbers were converted to a logarithmic scale (log 10) and then subjected to analysis of variance. Paired samples were compared using Student’s *t* test at a 95% confidence level (*p* < 0.05).

### 2.6 Microscopic Analysis of Bacteria

The scanning electron microscopy (SEM, JEOL model JSM-6380LV) was used to analyze the morphology of irradiated and nonirradiated bacteria. Approximately 200 μL of the solution containing bacteria was initially placed into a microtube Eppendorf with 1000 μL of glutaraldehyde and left for 3 h after completing the PDI assay. Next, 1000 μL of phosphate buffer solution (PBS) with a pH of 7.0 was introduced. Each sample was centrifuged at 1000 rpm during 5 min to remove the supernatant. PBS was used in the centrifugations three times, followed by ethanol concentrations of 25, 50, 60, 70, 80, 90% ethanol, and absolute ethanol. The resultant precipitate was dissolved in absolute ethanol and refrigerated. *E. coli* bacterial suspensions were immobilized on 1 cm × 1 cm glass substrates through overnight drying at room temperature. Afterward, a thin layer of gold was deposited onto the substrates using sputter-coating and affixed to SEM holders using conductive carbon tape. The micrographs were captured using 15 kV accelerating voltage, 10 μm spot size, and 8 mm working distance.

Confocal fluorescence images were acquired using the STELLARIS 5 microscope (Leica Microsystems, Germany). *E. coli* strains were subjected to PCPDTBT-NPs, following the procedure used in the aPDI assay. After that, 1 μL of the bacterial suspension was deposited onto a glass slide and covered with a coverslip. The images were acquired using a 63× oil immersion objective lens. To obtain the PCPDTBT-NPs confocal images, nanoparticle excitation was performed at 405 nm, collecting the emission in the 450–550 nm range. Parameters such as detector gain, pinhole, and scan speed were adjusted during image acquisition to optimize the signal-to-noise ratio.

## 3 Results and Discussion

### 3.1 Optical Characterization

**Figure 2:**
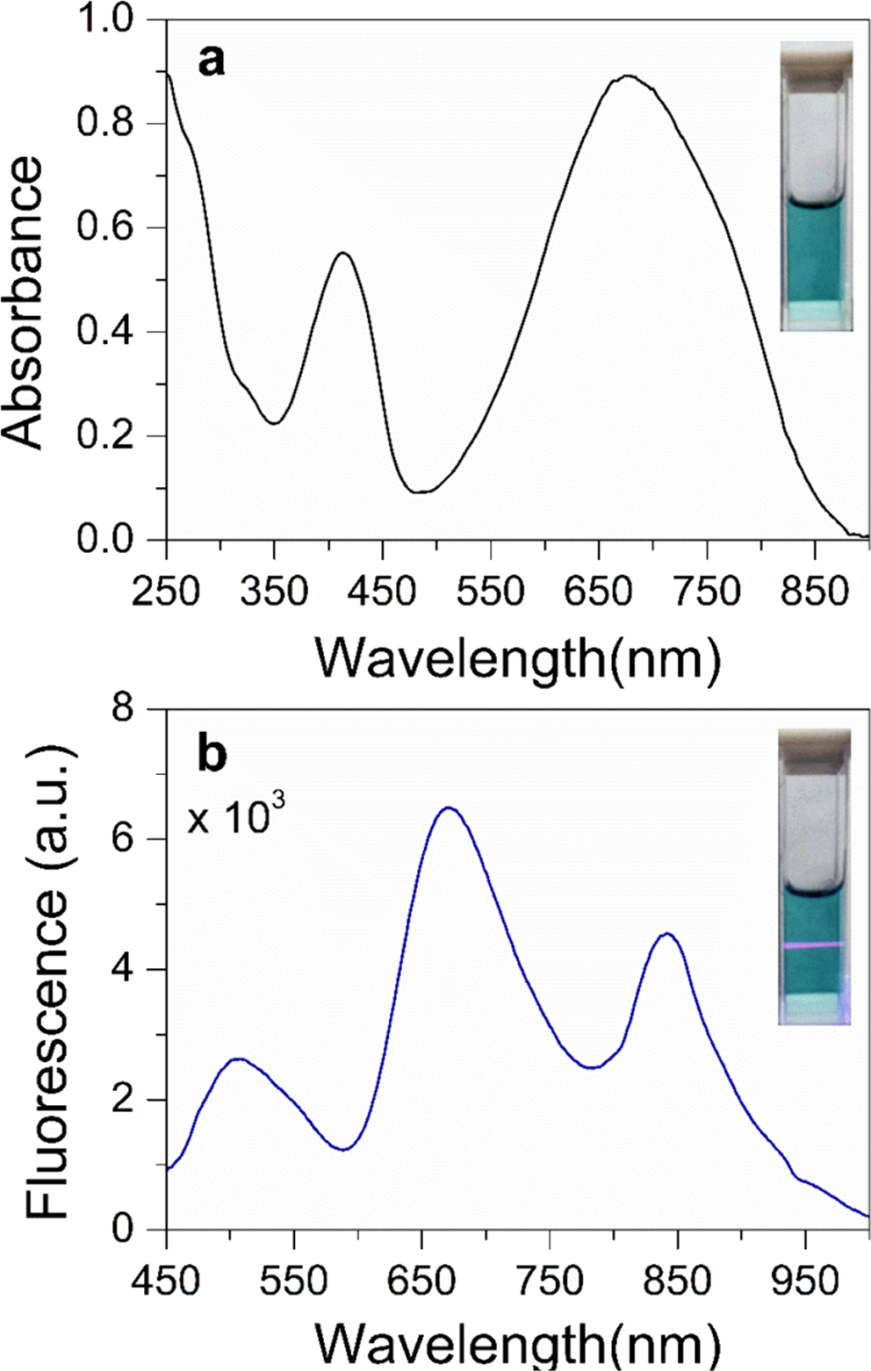
(a) UV–vis absorption spectrum of PCPDTBT-NPs and visual appearance of the solution; (b) Fluorescence spectrum of PCPDTBT-NPs under 405 nm excitation and image displaying the fluorescence in the aqueous solution with PCPDTBT at 17 μg·mL^–1^.

Figure 2 presents the absorbance and fluorescence spectra of PCPDTBT-NPs. The spectra of the PCPDTBT-NPs solution show broad absorption and fluorescence bands spanning in the UV to visible (250–900 nm) and visible to near-infrared (450–1050 nm) range, respectively. The data reveals two absorption bands with a maximum at around 405 and 680 nm (Figure 2a). The solution excited at 405 nm exhibits a fluorescence spectrum with three emission bands with maximum fluorescence at 505, 672, and 835 nm (Figure 2b). In contrast, as expected, only the emission centered at around 835 nm is observed by exciting the solution at 680 nm (data not shown). Their ability to absorb light in the visible region is a crucial property for bacterial photoinactivation, while their fluorescence makes them ideal building blocks for creating theranostic materials. PCPDTBT-NPs, extensively recognized as fluorescent probes,^33^ were previously investigated as promising fluorescent agents for assessing near-infrared wavelengths within an animal system.^29^ Their study also evaluated cytotoxicity and hemocompatibility, focusing on materials’ compatibility with animal blood. According to the paper, PCPDTBT-NPs successfully generated near-infrared images and exhibited low cytotoxicity and hemocompatibility,^29^ positioning them as reliable and biocompatible materials for various biomedical applications.

### 3.2 Characterization of PCPDTBT-NPs

ζ Potential is frequently used for surface charge characterization and quantitatively measuring the charge-induced colloidal stability in NPs dispersion. The sign of the ζ potential reveals whether the particle surface is predominantly positive or negative. Simultaneously, values exceeding +30 mV or below −30 mV imply favorable electrostatic stability.^34,35^ The ζ potential measured for PCPDTBT-NPs was −21.5 ± 0.3 mV, which falls short of the commonly accepted threshold of −30 mV for optimal colloidal stability. The negative charge exhibited by PCPDTBT-NPs suggests sufficient electrostatic repulsion to prevent significant agglomeration in the solution. The polydispersity index (PDI) of 0.38 ± 0.03 confirms that PCPDTBT-NPs are nearly monodisperse in the aqueous medium since PDI values between 0.1 and 0.7 indicate monodispersity.^36^

**Figure 3:**
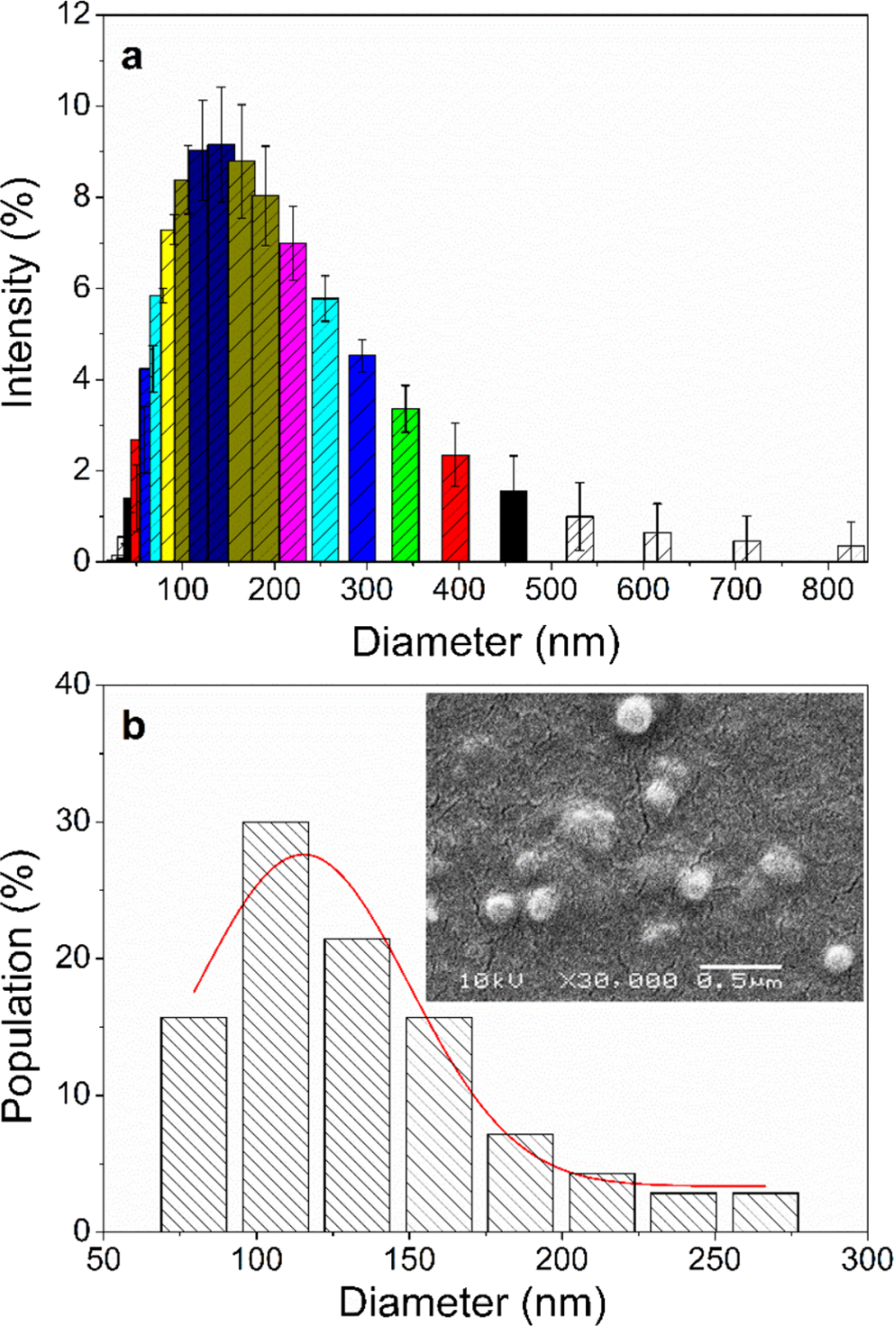
(a) Hydrodynamic diameter distribution, ζ potential, and polydispersity index (PDI) from dynamic light scattering measurements and (b) Particle diameter distribution and scanning electron micrography of PCPDTBT-NPs.

PCPDTBT-NPs exhibited a mean hydrodynamic diameter of 185 ± 77 nm (Figure 3a). This value reflects the particles’ effective size in solution, including their surrounding hydration layer and any possible interactions with solvent molecules. On the other hand, SEM offers a different perspective by capturing high-resolution images of individual particles in a dry state (Figure 3b). The mean diameter was calculated to be 122 ± 45 nm, highlighting the particles’ intrinsic geometric dimensions. Additionally, the SEM images show particles with a high degree of regularity in their spherical shapes.

**Figure 4:**
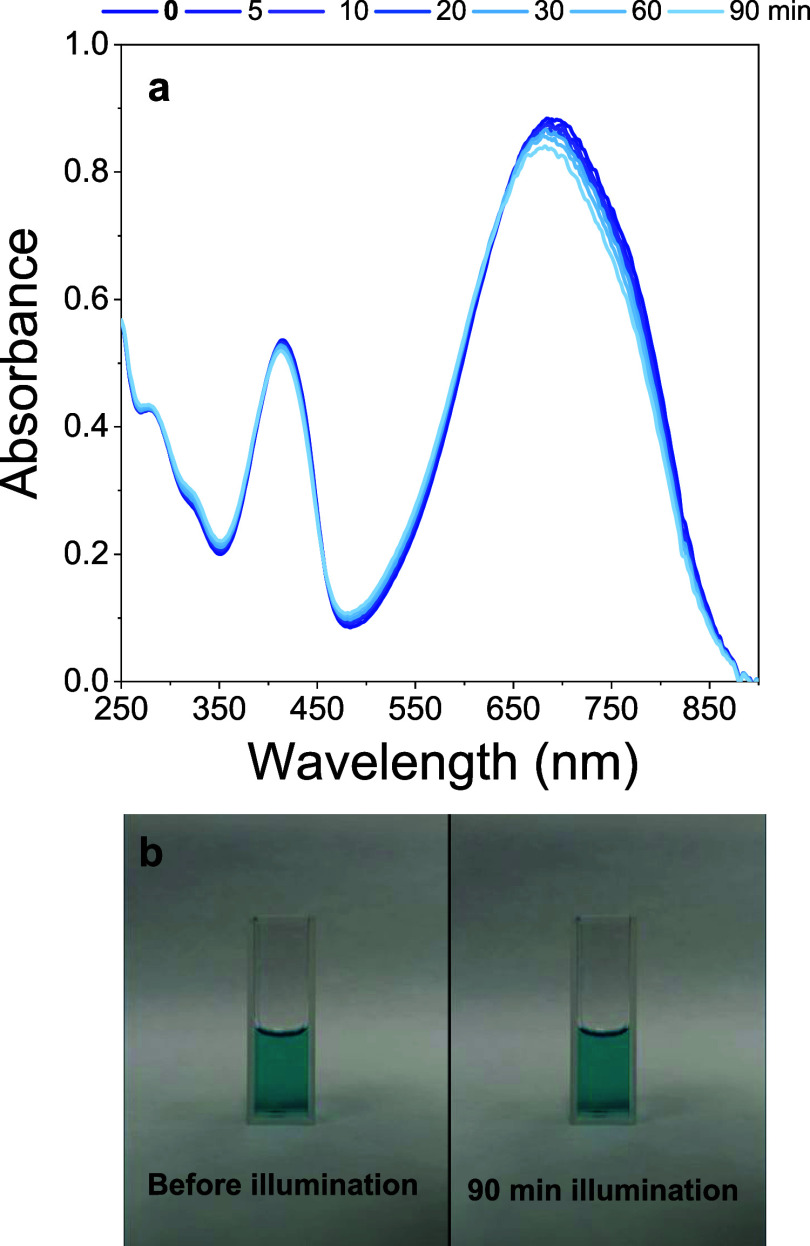
(a) UV–vis absorption spectrum of PCPDTBT-NPs as a function of the white light illumination and (b) visual appearance of the solution before and after 90 min of illumination.

**Figure 5:**
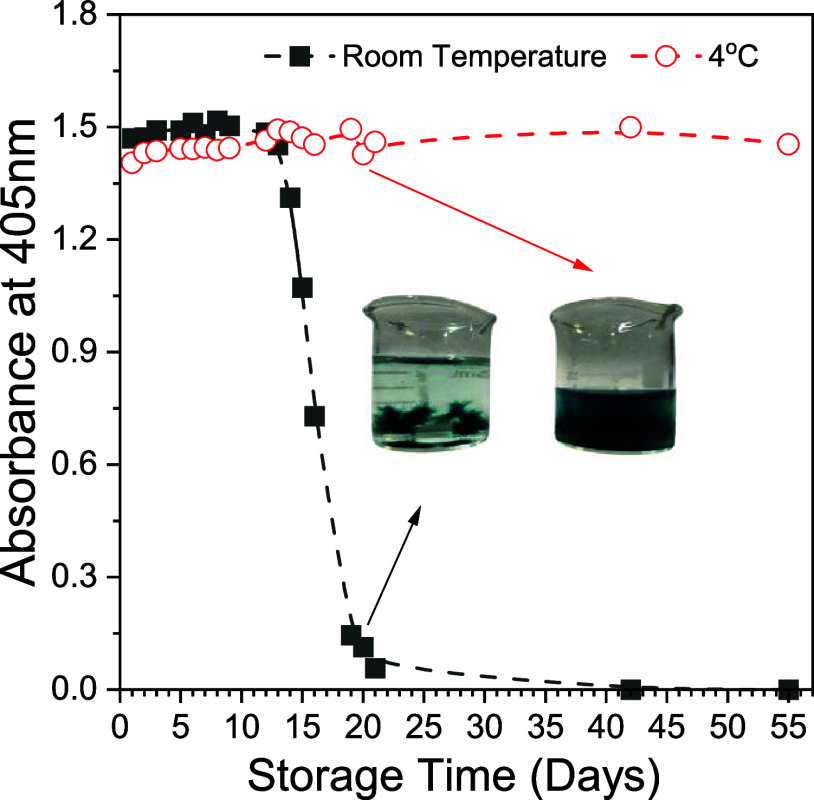
UV–vis absorption of PCPDTBT-NPs at 405 nm as a function of the storage time. Inset: visual appearance of the solution after 20 days.

In addition to the size and morphological characterization, the stored stability and photostability of the PCPDTBT-NPs solution were investigated. The nanoparticles are photostable when subjected to a light dose similar to those used during the aPDI procedure. Figure 4 shows no significative alteration in absorbance in the 250 to 650 nm range after 90 min of illumination (Figure 4). Additionally, PCPDTBT-NPs are very stable when stored in solution at 4 °C in the refrigerator. Although no polymer precipitation was observed during 8 weeks, it was observed after 3 weeks in the solution storage at room temperature (Figure 5).

### 3.3 Singlet Oxygen (^1^O_2_) Generation

**Figure 6:**
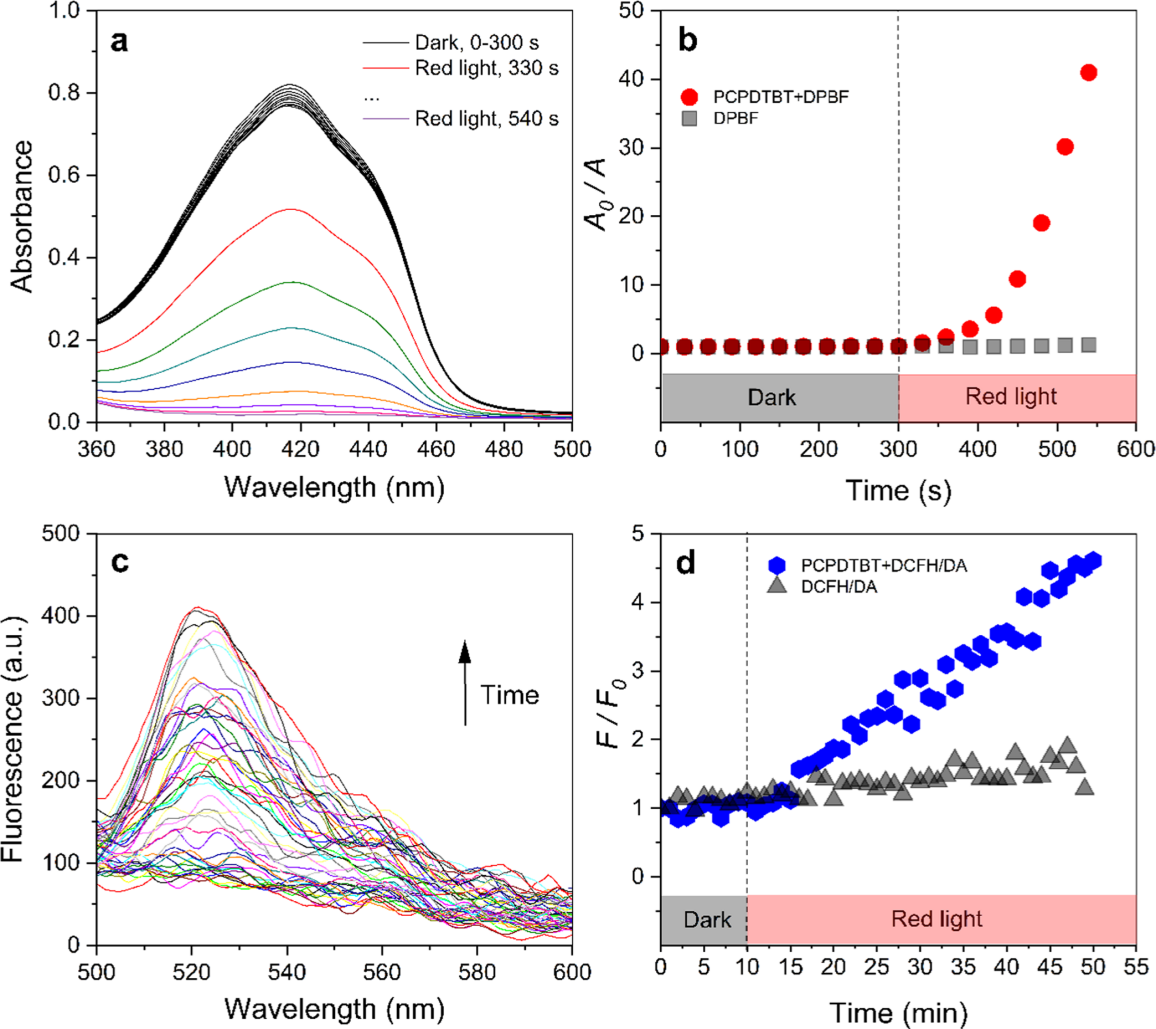
(a) Absorbance of DPBF (0.077 mM) in solution with and without PCPDTBT-NPs (8.5 μg·mL^–1^) as a function of time (625 nm irradiation, 3.5 mW), (b) *A*/*A*_0_ over time, where *A*_0_ and *A* is the time-zero absorption and the absorption at 415 nm, respectively, (c) Time-dependent fluorescence spectra of DCFH-DA (0.35 mM) in solution with and without PCPDTBT-NPs (8.5 μg·mL^–1^) under 625 nm irradiation (3.5 mW), and (d) *F*/*F*_0_ ratio over time, where *F*_0_ and *F* is the time-zero fluorescence and the fluorescence at 520 nm, respectively.

The time-dependent absorption spectra of DPBF solution with PCPDTBT-NPs are shown in Figure 6a. DPBF degraded upon 625 nm light in the presence of PCPDTBT-NPs, as evidenced by the reduction in its absorption. This observation provides compelling evidence for ^1^O_2_ production by PCPDTBT-NPs. During this process, DPBF is a complexing agent and forms an endoperoxide that decomposes into 1,2-dibenzoyl benzene (DBB), which does not absorb visible light.^37^Figure 6b supports this finding by presenting the initial and final absorption ratio at 415 nm. The data reinforces the observed reduction in DPBF absorption over time under red light, drawing attention to the photochemical capability of PCPDTBT-NPs to generate ^1^O_2_ during irradiation.

The fluorescence of the solution containing the nonfluorescent marker DCFH-DA and PCPDTBT-NPs showed a substantial enhancement over the exposure time to red light irradiation (Figure 6c). This rise suggests the formation of DCF, a highly fluorescent molecule, resulting from the interaction between the ROS produced by PCPDTBT-NPs under red light irradiation and the nonfluorescent marker DCFH-DA. Figure 6d highlights this increase in fluorescence, showing the evolution of the *F*/*F*_0_ ratio over time, where *F*_0_ represents the time-zero fluorescence, and *F* denotes the fluorescence at a specific time. It is valid to emphasize that there was no variation in the ratio value when the solution remained unexposed to irradiation (under dark conditions). This outcome is relevant considering the impact of ROS, which includes highly reactive oxygen radicals such as hydrogen peroxide (H_2_O_2_), nitric oxide, peroxynitrite, singlet oxygen (^1^O_2_), superoxide anions (O_2_^–^), and hydroxyl radicals (^•^OH). These radicals also play crucial roles in the photodynamic inactivation of bacteria.^38^

### 3.4 Photoinactivation Assay

**Figure 7:**
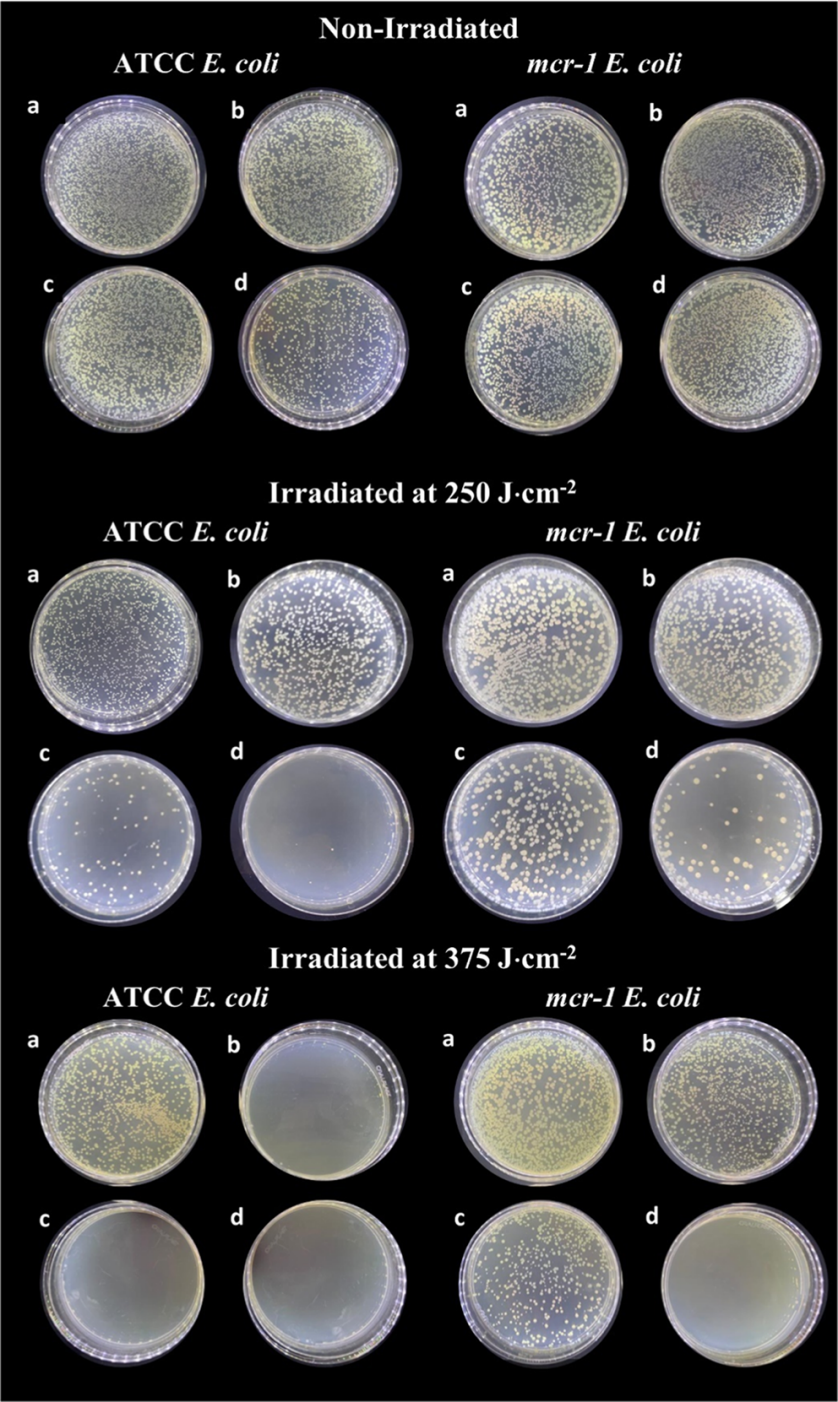
Growth of *E. coli* (ATCC 25922) and *mcr-1* positive *E. coli* (CCBH 23595) colonies with (a) 0.0, (b) 4.25, (c) 8.5, and (d) 17 μg·mL^–1^ of PCPDTBT-NPs on plate count agar. The bacterial media were nonirradiated and white light irradiated with energy doses of 250 and 375 J·cm^–2^ for 60 and 90 min, respectively.

Figure 7 displays representative images of *E. coli* colonies (strains ATCC and *mcr-1*) for the tested PCPDTBT-NPs concentrations and irradiation conditions: nonirradiated (dark) and white light irradiation from the combination of 625, 450, and 525 nm with energy doses of 250 and 375 J·cm^–2^ for 60 and 90 min, respectively.

The results show a difference in the growth of *E. coli* colonies submitted to PCPDTBT-NPs and white light irradiation compared to those not irradiated. Specific concentrations of PCPDTBT-NPs in the growth medium significantly reduced the colony growth of *E. coli* exposed to white light illumination. Additionally, PCPDTBT-NPs alone could not inhibit the development of the nonirradiated *E. coli* colonies.

PCPDTBT-NPs at a concentration of 17 μg·mL^–1^ exhibited photoantimicrobial properties by effectively inactivating plasmid-mediated colistin-resistant *E. coli*. The antibiotic-susceptible strain showed lower susceptibility to antimicrobial photodynamic inactivation (aPDI). This observation is consistent with prior studies that have reported reduced vulnerability of antibiotic-resistant bacteria to aPDI when employing other photosensitizers.^29,30^ For instance, a study with approximately 400 clinical samples of methicillin-resistant *Staphylococcus aureus* (MRSA) and methicillin-susceptible *S. aureus* (MSSA) found that MRSA exhibits greater tolerance to aPDI.^39^ However, the enhanced tolerance was not attributed to the *mecA* gene. Instead, it could be linked to the bacterium′s defense mechanisms against oxidative stress and efflux pumps that hinder photosensitizer absorption.^39^ Kashef et al. also presented similar findings by photoinactivating susceptible and resistant strains of *E. coli* and *S. aureus* using two phenothiazine photosensitizers, methylene blue and toluidine blue. Their results revealed a lower tolerance to aPDI of susceptible strains than the resistant strains.^40^

**Figure 8:**
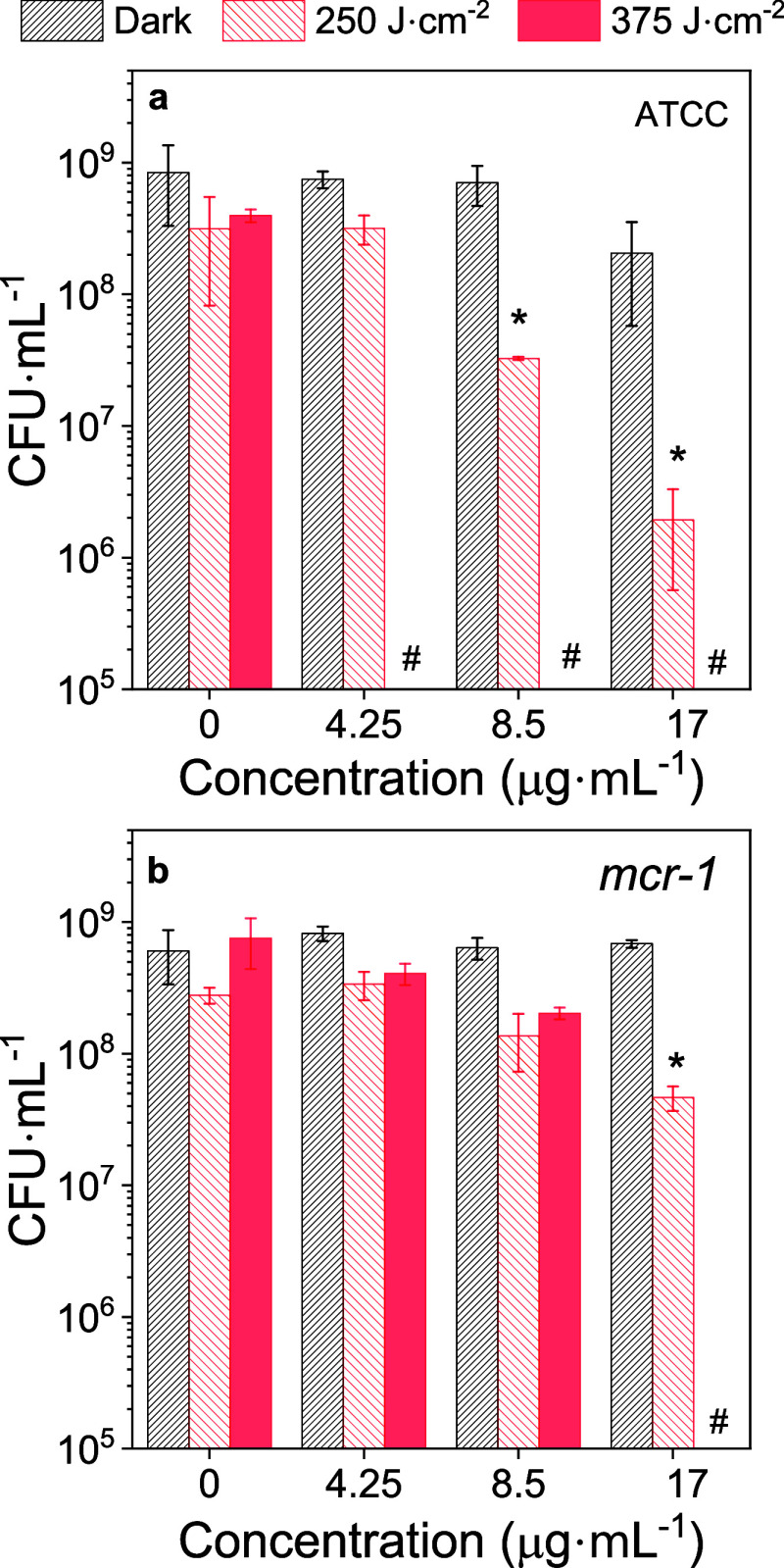
Mean CFU·mL^–1^ of *E. coli* (*ATCC*) and *mcr-1* positive *E. coli* (CCBH 23593) subjected to PCPDTBT-NPs. The irradiated group underwent white light illumination with energy doses of 250 and 375 J·cm^–2^. The asterisks (*) indicates a significant difference at a 95% confidence level (*p* < 0.05), whereas the hash symbol (#) denotes that no bacterial colony was observed.

The mean CFU·mL^–1^ values in Figure 8 indicate the successful use of PCPDTBT-NPs as photosensitizers against ATCC and *mcr-1* strains of *E. coli*. There was a reduction in CFU·mL^–1^ by exposing both strains to a PCPDTBT-NPs concentration of 17 μg·mL^–1^ and 250 J·cm^2^ irradiation. The ATCC *E. coli* had a more substantial decline in CFU·mL^–1^ by 97% than the *mcr-1* strain, which showed a fall of 83%. A bactericidal effect succeeded with a dose of 375 J·cm^–2^ at a concentration of 4.25 μg·mL^–1^ against ATCC and 17 μg·mL^–1^ against the *mcr-1* strain. Consequently, the difference in the responses of antibiotic-susceptible and–resistant strains to aPDI underlines the significance of considering strain-specific characteristics when designing and implementing photoantimicrobial strategies.

PCPDTBT-NPs have been widely applied in the biomedical field as fluorescent markers^41,42^ and photoresponsive agents for heat generation.^26^ PCPDTBT-containing hybrid nanoparticles possess aPDI capabilities and function as coagents for diagnostic imaging or photothermal therapy. In this context, the aPDI action is promoted by a photosensitizer associated with PCPDTBT.^27,43,44^ Here, it is reported for the first time that nanoparticles composed solely of PCPDTBT can be utilized as photosensitizers to reduce bacterial growth employing aPDI.

### 3.5 Bacterial Microscopy Analysis

**Figure 9:**
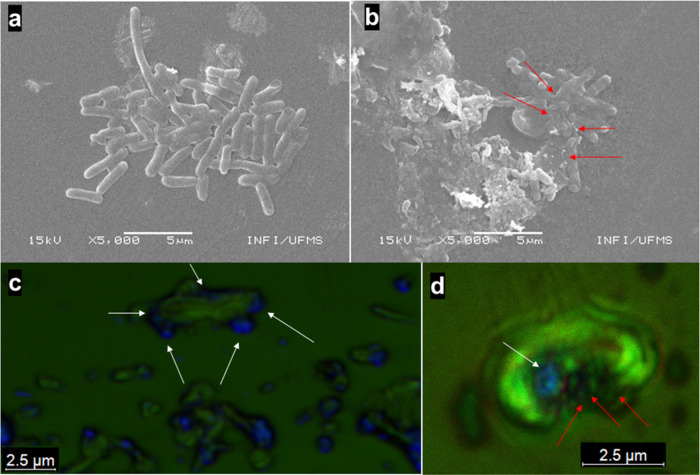
Representative SEM image of *E. coli* exposed to PCPDTBT-NPs at a concentration of 17 μg·mL^–1^: (a) nonilluminated and (b) after white light illumination (250 J·cm^–2^). Red arrows show partial or complete damage to the cell wall. Confocal fluorescence image of *E. coli* subjected to PCPDTBT-NPs at 17 μg·mL^–1^: (c) nonilluminated and (d) after white light illumination (250 J·cm^–2^). The blue dots represent the PCPDTBT-NPs fluorescence.

SEM images show the rod shapes of *E. coli* with an average size ranging from approximately 2 to 3 μm (Figure 9a). The micrographs depict the pristine, intact morphology of nonirradiated *E. coli* bacteria. In contrast, the photodynamic inactivation process produces notable changes, as seen in Figure 9b, causing partial or complete damage to the bacterial cell wall when exposed to a light dose of 250 J·cm^–2^. The observed damages have the potential to induce bacterial lysis, thereby effectively halting bacterial growth. Confocal fluorescence images confirmed the preferential localization of PCPDTBT-NPs on bacterial membranes (Figure 9c) and the bacterial cell wall damage promoted for aPDI after white light illumination (Figure 9d).

## 4 Conclusions

The effectiveness of PCPDTBT-NPs as standalone photosensitizers for inactivating multiresistant *mcr*-1-positive *E. coli* has been proven without the need for other agents normally utilized. This breakthrough was achieved using white light from RGB LEDs and without any dark toxicity concerns, marking a departure from conventional NIR radiation. The effectiveness of PCPDTBT-NPs as photosensitizers, operating through type I and type II photodynamic mechanisms, underscores their potential as candidates for further *in vivo* exploration. This development paves the way for innovative LED-based antimicrobial strategies, opening new research and applications.

## Data Availability

Data will be made available on request.
